# Quetiapine: Relatively safe in overdose?

**DOI:** 10.4103/0019-5545.49456

**Published:** 2009

**Authors:** Surendra K. Mattoo, Ruchita Shah, R. Rajagopal, Partha Sarathy Biswas, Shubh Mohan Singh

**Affiliations:** Department of Psychiatry, Postgraduate Institute of Medical Education and Research, Chandigarh - 160 012, India

**Keywords:** Overdose, quetiapine, schizophrenia, psychosis, deliberate self harm

## Abstract

Compared to other antipsychotics quetiapine has been reported to be relatively safer in overdose. We report a case with paranoid schizophrenia who attempted suicide with 1400 mg of quetiapine and manifested drowsiness, supraventricular tachycardia (167/minute) and minimal ST depression in leads V1 to V6 on ECG; all other physiological parameters were normal. Gastric lavage, lorazepam 2mg i/v to control agitation, and 14-hour observation in emergency ended in she being sent home. Subsequently she was successfully managed with ECTs, and quetiapine 600mg and risperidone 6mg daily. This report tends to support the literature suggesting quetiapine as a relatively-safer-in-overdose antipsychotic, and preferable in medication-overdose-suicidal-risk cases.

## INTRODUCTION

Quetiapine is being increasingly used as a first line antipsychotic. In overdose it is reported to be safer than the traditional antipsychotics.[1–6] We present a case to highlight this reported overdose safety of quetiapine.

## CASE REPORT

Ms. M. was a 23-year-old graduate with no family or past history of significant medical or psychiatric illness. Her psychiatric illness of insidious onset and continuous course of 3 years was characterized by muttering and laughing to self, and undressing in public without reason, social withdrawal, irritability and anger outbursts, delusions of reference and persecution, and auditory hallucinations of commanding type; but no significant affective or cognitive symptoms. Two years back for no reason but with a serious suicidal intent she had consumed toilet-cleansing liquid without any medical sequel. Since then she was under psychiatric treatment, which included 5 ECTs, and for the last 9 months tablets quetiapine 400 mg, chlorpromazine 150 mg, risperidone 4 mg and clonazepam 2 mg daily; with partial improvement (GAF score-30) despite good compliance ensured by the family.

Following an anger outburst she consumed 14 tablets of quetiapine 100 mg and within one hour she was brought to our emergency services. There was no history of any other concurrent drug overdose. Physical examination was normal except for drowsiness and tachycardia (140/minute); there was no respiratory depression. Mental status examination showed good orientation, commanding auditory hallucinations, marked hostility, and anger outbursts. She gave no reason for the overdose. Routine hematology, biochemistry, and chest x-ray were normal; ECG showed supraventricular tachycardia (167/minute) and minimal ST depression in leads V1 to V6. Gastric lavage was done within 2 hours of overdose; lorazepam 2 mg i/v was used once to control agitation. After 14-hour observation in emergency she was sent home.

The next day at psychiatric outpatient follow-up quetiapine was increased to 600 mg daily (risperidone and clonazepam were not prescribed). Three weeks later another suicidal attempt by wrist slashing forced her admission to the psychiatry ward. A repeat ECG showed supraventricular tachycardia (152/minute); there were no other cardiac symptoms/signs, and hematology and biochemistry were all normal. The cardiologist opined ‘medication-side-effect’ and advised no active intervention. The tachycardia subsided over the next one week. With no improvement with over 8 weeks of quetiapine 600 mg daily, 9 alternate-day ECTs were given. Lack of further improvement and her past best response to quetiapine-risperidone combination led to re-induction of risperidone, increased over 3 weeks to 6 mg/day. Non-pharmacological measures (activity scheduling, reinforcement regime/token economy) were also added. Over the next 9 weeks she showed significant improvement, to GAF score of 76. Overall, her recovery from overdose was uneventful and did not adversely affect reinstitution of quetiapine or risperidone and successful plastic surgery for repair of median nerve, damaged during wrist slashing. With good treatment compliance for the last one and a half year she has maintained her improvement. During follow up 4 weeks back her cardiac examination and repeat ECG showed no abnormality except for sinus tachycardia (100/minute).

## DISCUSSION

Overdose is broadly defined as inadvertent or deliberate consumption of larger than the usual dose of any substance, often leading to serious toxic reactions or death.[8] Our case satisfies this definition of overdose. Quetiapine is reportedly well tolerated in therapeutic doses and relatively safe in overdoses. The overdoses are characterized by hypotension, tachycardia, and somnolence as predicted by its known alpha-adrenergic receptor and histamine receptor blockade.[3] In a series of 14 cases tachycardia and somnolence were cited as the main clinical symptoms; the severity not being associated with either high serum concentration or the reported ingested dose of quetiapine.[5] Another series of 45 cases of Quetiapine overdose highlighted central nervous system depression and sinus tachycardia.[4] In large overdoses, patients may require intubation and ventilation for associated respiratory depression.[4] A case report documented prolonged QT interval following approximately 10,000 mg of quetiapine and co-medication with fluvoxamine which being an enzyme inhibitor might have increased the propensity of toxicity.[1] The clinical significance of prolonged QTC in quetiapine overdose is unclear but likely to be a result of an overcorrection caused by the tachycardia. This is evident from the series reporting that out of seven overdose (>900 mg) incidents, none was lethal and only one had a heart block.[7] Lastly, a case report documented acute respiratory distress syndrome,[6] while another reported death at a serum quetiapine level of 18,300 ng/ml.[2]

There is no specific antidote, and quetiapine overdose is managed by appropriate supportive measures. These include gastric lavage and administration of activated charcoal and a laxative, maintaining airway and ensuring adequate ventilation and oxygenation, and continuous cardiovascular - including electrocardiographic – monitoring.

In our case there was evidence of drowsiness, and supraventricular tachycardia and ST segment depression on ECG. She could be managed with only gastric lavage and observation in the emergency. Later she could be continued on quetiapine and quetiapine-risperidone co-prescription without any adverse effects. Though the overdose was not very high (only 1400mg), our case supports the conclusion that more often than not quetiapine overdoses are mild and thus quetiapine may be an antipsychotics of choice in patients with risk of medication based suicidal attempts.
